# Risk of tuberculosis in patients with spondyloarthritis: data from a centralized electronic database in Hong Kong

**DOI:** 10.1186/s12891-020-03855-5

**Published:** 2020-12-10

**Authors:** Natalia Chu-Oi Ciang, Shirley Chiu Wai Chan, Chak Sing Lau, Eva Tsz Fung Chiu, Ho Yin Chung

**Affiliations:** 1 Division of Rheumatology, Department of Medicine, Queen Elizabeth Hospital, Hong Kong, Hong Kong; 2 Division of Rheumatology and Clinical Immunology, The University of Hong Kong, Hong Kong, Hong Kong

**Keywords:** Spondyloarthritis, Tuberculosis, Disease modifying anti-rheumatic drugs, Glucocorticoid, Infliximab

## Abstract

**Background/ objective:**

Tuberculosis (TB) is one of the most infectious comorbidities in spondyloarthritis (SpA). Our goals were to determine the crude incidence rate of and risk factors for TB in SpA.

**Method:**

Clinical data of 2984 patients with SpA from 11 rheumatology centres were reviewed. This included demographics, duration of follow-up, comorbidities including diabetes, chronic kidney disease, chronic heart disease, chronic lung disease, stroke and malignancies, date of diagnosis of tuberculosis, use of non-steroidal anti-inflammatory drugs, duration of glucocorticoid therapy for more than 6 months, conventional (cDMARD) and biological (bDMARD) disease modifying anti-rheumatic drug therapies. Crude incidence rates were reported. Cox regression models were used to determine the risk factors for TB in patients with SpA.

**Results:**

Forty-three patients had TB, of which 4 (9.3%) were extra-pulmonary. The crude incidence rate of TB was 1.57 in patients with SpA, compared with 0.58 in the general population in Hong Kong. Independent risk factors identified from the multivariate Cox regression model were: alcohol use (HR 2.62; *p* = 0.03), previous TB (HR 13.62; *p* < 0.001), chronic lung disease (HR 3.39; *p* = 0.004), duration of glucocorticoid therapy greater than 6 months (HR 3.25; *p* = 0.01) and infliximab therapy (HR 5.06; *p* < 0.001). Age was associated with decreased risk (HR 0.93; *p* < 0.001).

**Conclusion:**

Incidence of TB was higher in patients with SpA. Glucocorticoid therapy beyond 6 months and infliximab therapy increased the risk of TB. Rheumatologists should avoid prolonged use of glucocorticoids and consider DMARDs other than infliximab in the treatment of at-risk patients.

## Introduction

Spondyloarthritis (SpA) is a spectrum of inflammatory rheumatic diseases comprised of ankylosing spondylitis (AS), psoriatic arthritis (PsA), enteropathic arthritis associated with inflammatory bowel disease (IBD), reactive arthritis, undifferentiated spondyloarthritis (uSpA) and human leucocyte antigen B27 (HLA-B27) associated uveitis. SpA leads to impairments in quality of life, work, leisure and daily activity [1], and is associated with many comorbidities including infection [2].

Tuberculosis (TB) is one of the most important infectious comorbidities in SpA. TB leads the global burden of morbidity and mortality, causing 1.4 million deaths annually, with the majority in Africa and Asia [3]. Previous studies have shown increased rates of TB in AS [4, 5], PsA [6] and other subtypes of SpA [7], and in patients on tumour necrosis factor inhibitor (TNFi) therapy [8, 9]. South Korean data found more TB in AS patients on TNFi therapy, with incidence rate ratios of 4.87 compared with other drug treatments [5], and 6.4 compared with the general population [10].

The crude incidence rate of TB in the general population in Hong Kong was 58.1 per 100,000 in 2018 [11], much higher than in western populations. TNFi therapy is known to trigger reactivation of latent TB [12, 13], yet conventionally used to treat SpA. Biologic drugs other than TNFi are increasingly prescribed due to lower risk of TB [14].

The Hong Kong Society of Rheumatology guidelines for screening and treatment of active and latent TB prior to starting biologics [15], though adopted by local clinicians for many years, is not foolproof in its prevention in patients with SpA [16]. The objectives of this study are to determine the crude incidence rate of and risk factors for TB in SpA.

## Method

Clinic data were retrieved from the Clinical Management System (CMS) of the Hospital Authority, a centralized electronic database of medical records in all public hospitals in Hong Kong. All patients with a diagnosis of SpA were identified and reviewed by the author (HYC), a specialist in Rheumatology and Fellow of the Royal College of Physicians of Edinburgh and Hong Kong College of Physicians. These included cases of AS, PsA, IBD-associated SpA, reactive arthritis, uSpA, and HLA-B27 associated uveitis from all eleven rheumatology centers in Hong Kong (Queen Mary Hospital, Grantham Hospital, Tung Wah Hospital, Alice Ho Miu Ling Nethersole Hospital, Caritas Medical Centre, Kwong Wah Hospital, Queen Elizabeth Hospital, Pamela Youde Nethersole Eastern Hospital, Pok Oi Hospital, Prince of Wales Hospital and Tseung Kwan O Hospital). Data were collected from Feb 1994 to June 2019 by one clinician (HYC) and randomly scrutinized by another (SCWC).

Clinical data retrieved were age, sex, smoking status, alcohol use, dates of first and last follow-up, comorbidities (including diabetes, chronic kidney disease, chronic heart disease, chronic lung disease, stroke and malignancy), DMARD therapy, glucocorticoid therapy greater than 6 months, and other immunosuppressive states (chemotherapy, immunosuppressant therapy other than DMARDs, congenital and acquired immunodeficiency [including human immunodeficiency viruses [HIV] infection]). Chronic renal impairment was defined as chronic kidney disease stage 3 or above [17]. Chronic lung disease included asthma, chronic obstructive airway disease, bronchiectasis, and interstitial lung disease. Chronic heart disease included ischemic heart disease, congenital heart disease, heart failure, valvular heart disease, septal defect, and arrhythmia. Cerebrovascular accident included both ischemic and hemorrhagic stroke. Medication histories included dates of initiation and discontinuation of the following conventional disease-modifying antirheumatic drugs (c-DMARDs): sulphasalazine, methotrexate and leflunomide; and biologic disease-modifying antirheumatic drugs (b-DMARDs): etanercept, infliximab, adalimumab, golimumab, certolizumab, secukinumab and ustekinumab. The primary outcome measure was the presence of new onset of TB after the diagnosis of SpA. Past history of TB was also recorded.

### Duration of follow-up

Duration of follow up was defined as the time between first assessment at the rheumatology clinic and one of the following endpoints: first admission due to tuberculosis, death, last day of follow up, or end of study.

### DMARD therapy

In patients with new onset TB, concurrent DMARD therapy was defined as having been prescribed within a month prior to its diagnosis. In patients without TB, DMARD therapy was defined as having been prescribed at any time within the follow up period.

### Statistical analyses

Patients with and without TB were compared using the student t-test for continuous variables and the Pearson’s chi-square test for categorical variables.

Crude incidence rates of TB were described as per 1000 patient-years. Univariate Cox regression analyses were used to screen the following risk factors for TB: age [18], male gender [19], smoking [20] and alcohol use [20], diabetes mellitus (DM) [19], malignancy, chronic kidney disease, chronic lung disease, chronic heart disease, stroke, previous history of TB [18, 21] and other immunosuppressive states. Duration of follow-up was considered in the time variable of individual analyses. Significant independent variables with a *p*-value < 0.1 were included in the multivariate Cox regression model using enter mode. Results were reported as HR and 95% confidence interval (CI). A *p*-value of less than 0.05 was defined as statistically significant. All statistics were performed using the International Business Machines Corporation Statistical Package for the Social Sciences (IBM SPSS) package 25.0. Listwise deletion was performed in the analyses (missing values not included).

## Results

Out of 2969 patients with SpA in the study, 1940 (65.3%) had AS, 642 (21.6%) had PsA, 47 (1.6%) had IBD-associated SpA, and 6 (0.2%) had reactive arthritis. Baseline characteristics are shown in Table 1. The group of 43 patients with TB was characterized by male predominance, younger age, shorter duration of follow up, smoking and alcohol use, past history of TB, chronic lung disease, and cerebrovascular accident. More patients in this group were treated with glucocorticoid and infliximab (Table 2). No statistically significant differences were found with psoriasis, IBD, DM, chronic kidney disease, chronic heart disease and other immunosuppressive states. There was a tendency for malignancy in the group with TB (Table 1).

**Table 1:** Baseline characteristics of SpA patients with and without TB

	SpA with TB	SpA without TB	*P* value	Total
Chinese ethnicity	43/43 (100%)	2896/2926 (99.0%)	0.51	2939/2969 (99.0%)
Male sex	36/43 (83.7%)	1993/2926 (68.1%)	0.03	2029/2969 (69.3%)
Age (years)	43.5 ± 16.2	49.9 ± 14.6	0.01	49.8 ± 14.6
Duration of follow up (years)	12.6 ± 5.5	9.2 ± 5.9	< 0.001	9.2 ± 1.2
Radiographic sacroiliitis	34/42 (81.0%)	1906/2784 (68.5%)	0.08	1305/1707 (76.4%)
HLA-B27 status	13/16 (81.3%)	1292/1691 (76.4%)	0.65	1940/2826 (68.6%)
Smoking	19/43 (44.2%)	856/2875 (29.8%)	0.04	875/2918 (30.0%)
Alcohol use	8/43 (18.6%)	232/2875 (8.1%)	0.01	240/2918 (8.1%)
Past history of TB	9/43 (20.9%)	69/2926 (2.4%)	< 0.001	78/2969 (2.6%)
psoriasis	6/43 (14.0%)	636/2926 (21.7%)	0.22	642/2969 (21.6%)
IBD	1/43 (2.3%)	46/2926 (1.6%)	0.69	47 (2969) (1.6%)
ReA	0/43 (0.0%)	6/2926 (0.2%)	0.77	6/2969 (0.2%)
Diabetes Mellitus	4/43 (9.3%)	265/2926 (9.1%)	0.96	269/2969 (9.1%)
Chronic kidney disease	4/43 (9.3%)	183/2926 (6.3%)	0.41	187/2969 (6.3%)
Malignancy	4/43 (9.3%)	111/2926 (3.8%)	0.06	115/2969 (3.9%)
Chronic lung disease	8/43 (18.6%)	90/2926 (3.1%)	< 0.001	98/2969 (3.3%)
Chronic heart disease	4/43 (9.3%)	190/2926 (6.5%)	0.46	194/2969 (6.5%)
Cerebrovascular accident	4/43 (9.3%)	99/2926 (3.4%)	0.04	103/2969 (3.5%)
Other immunosuppressive states	1/43 (2.3%)	56/2926 (1.9%)	0.85	57/2969 (1.9%)

*SpA* Spondyloarthritis; *TB* Tuberculosis; *IBD* Inflammatory bowel disease; *ReA* Reactive arthritis

**Table 2:** NSAID, glucocorticoid, and DMARD therapy in SpA with and without TB

	SpA with TB	SpA without TB	*P* value
NSAIDs	43/43 (100.0%)	2783/2926 (95.1%)	0.14
glucocorticoid therapy > 6 months	6/43 (14.0%)	148/2926 (5.1%)	0.01
DMARDs	29/43 (67.4%)	1831/2926 (62.6%)	0.51
cDMARDs	21/43 (48.8%)	1609/2926 (55.0%)	0.42
sulfasalazine	16/43 (37.2%)	1253/2926 (42.8%)	0.46
methotrexate	9/43 (20.9%)	763/2926 (26.1%)	0.45
leflunomide	0/43 (0.0%)	156/2926 (5.3%)	0.12
bDMARDs	17/43 (39.5%)	709/2926 (24.2%)	0.02
TNFi	17/43 (39.5%)	666/2926 (22.8%)	0.001
infliximab	10/43 (23.3%)	98/2926 (3.3%)	< 0.001
etanercept	2/43 (4.7%)	268/2926 (9.2%)	0.31
adalimumab	5/43 (11.6%)	235/2926 (8.0%)	0.39
golimumab	0/43 (0.0%)	196/2926 (6.7%)	0.08
certolizumab	0/43 (0.0%)	39/2926 (1.3%)	0.45
secukinumab	0/43 (0.0%)	69/2926 (2.4%)	0.31
ustekinumab	0/43 (0.0%)	19/2926 (0.6%)	0.60

*NSAID* Non-steroidal anti-inflammatory drug; *SpA* Spondyloarthritis; *TB* Tuberculosis; *DMARDs* Disease modifying anti-rheumatic drugs; *cDMARDs* Conventional disease modifying antirheumatic drugs; *bDMARDs* Biologic disease modifying antirheumatic drugs; *TNFi* Tumour necrosis factor inhibitor

One case of TB was recorded before the implementation of pre-biologic therapy screening for latent TB in 2001. This case had not received any biologic therapy.

### Crude incidence rates of TB infection

This cohort was characterized by long duration of follow-up, with an overall 27,308.4 patient-years in SpA. Subgroup analyses showed 18,204.2 and 9104.2 patient-years respectively for SpA on DMARDs and not on DMARDs. Crude incidence rates of TB in the three groups above were higher than in the general population in 2018 [11]. Results are shown in Table 3. Crude incidence rates remained high even after adjusting for age and sex (Table 3). Most of the female patients with TB had predisposing risk factors. Out of 7, 2 were smokers, 2 had a previous history of TB, 2 were on infliximab, and 3 were on long term glucocorticoid therapy.

**Table 3:** Crude incidence rates of TB

	Patients with SpA		General population (11)	
Patient-years	27,308.4			
Number of events	43			
Incidence per 1000 patient-years	0.64		0.54	
	on DMARD	not on DMARD		
Patient-years	18,204.2	9104.2		
Number of events	29	14		
Incidence per 1000 patient-years	0.62	0.65	0.54	
	Male	Female	Male (age adjusted)	Female (age adjusted)
Patient-years	18,693.8	8614.5		
Average age	49	52		
Number of events	36	7		
Incidence per 1000 patient-years	0.52	1.23	0.48	0.41

*TB* Tuberculosis; *SpA* Spondyloarthritis; *DMARD* Disease modifying antirheumatic drug

### Sites of TB infection

**Fig. 1:**
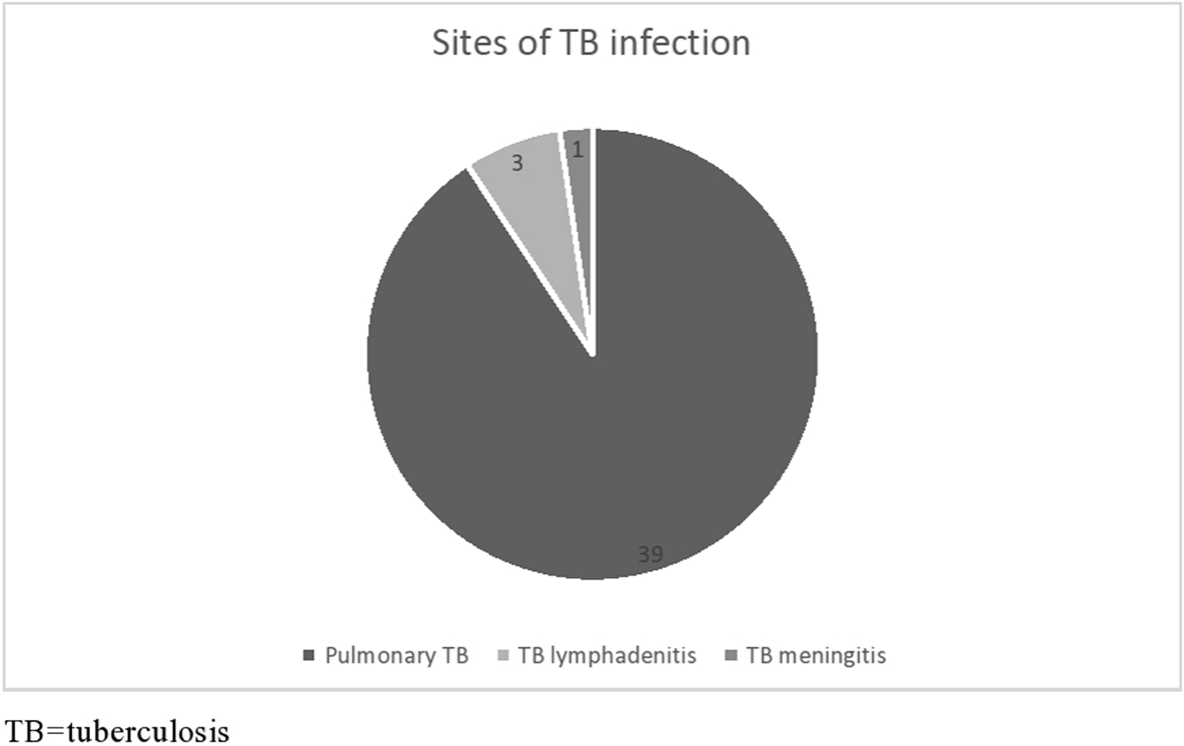
Sites of TB infection

In the group with TB, 39 (91%) had pulmonary, and 4 (9.3%) had extra-pulmonary TB. Three had TB lymphadenitis and 1 had TB meningitis (Fig. 1).

### Risk factors for TB

Univariate Cox regression models screened for risk factors for TB in patients with SpA. Covariates with a *p*-value < 0.10 were age (HR 0.93; *p* = 0.01), male gender (HR 2.29; *p* = 0.05), smoking (HR 1.74; *p* = 0.07), alcohol use (HR 2.29; *p* = 0.04), previous history of TB (HR 6.88; *p* < 0.001), chronic lung disease (HR 4.48; *p* < 0.001), duration of glucocorticoid therapy greater than 6 months (HR 2.21; *p* = 0.03), infliximab therapy (HR 5.08; *p* < 0.001).

The multivariate Cox regression model showed that younger age, alcohol use, previous history of TB, chronic lung disease, duration of glucocorticoid therapy greater than 6 months and infliximab therapy were independent risk factors for TB in SpA. Results are shown in Table 4.

**Table 4:** Univariate and multivariate cox regression models of tuberculosis in SpA

	Univariate regression		Multivariate logistic regression	
Characteristic	Hazard Ratio (95% CI)	*P* value	Hazard Ratio (95% CI)	*P* value
Age (years)	0.93 (0.91–0.95)	< 0.001	0.94 (0.91–0.96)	< 0.001
Male sex	2.29 (1.02–5.15)	0.05	1.88 (0.79–4.50)	0.16
Smoking	1.74 (0.95–3.19)	0.07	1.19 (0.60–2.35)	0.62
Alcohol use	2.29 (1.06–4.94)	0.04	2.44 (1.03–5.80)	0.04
History of psoriasis	0.51 (0.21–1.20)	0.12		
History of IBD	1.43 (0.20–10.37)	0.73		
DM	0.72 (0.26–2.03)	0.54		
Past history of TB	6.88 (3.28–14.41)	< 0.001	5.92 (2.52–13.94)	< 0.001
CKD	0.89 (0.32–2.51)	0.83		
CLD	4.48 (2.07–9.72)	< 0.001	3.81 (1.60–9.06)	0.002
Malignancy	2.07 (0.74–5.80)	0.17		
CHD	0.88 (0.31–2.47)	0.81		
Other immunosuppressive states	0.95 (0.13–6.89)	0.96		
History of CVA	1.46 (0.52–4.09)	0.48		
Glucocorticoid therapy > 6 months	2.21 (0.93–5.25)	0.03	2.60 (1.01–6.70)	0.05
Sulfasalazine	0.63 (0.34–1.16)	0.14		
Methotrexate	0.57 (0.27–1.19)	0.13		
Lefluonomide	0.05 (0.00–13.72)	0.29		
Infliximab	5.08 (2.49–10.34)	< 0.001	3.94 (1.82–8.53)	< 0.001
Etanercept	0.57 (0.14–2.36)	0.44		
Adalimumab	1.83 (0.72–4.67)	0.21		
Certolizumab	0.05 (0.00–32,169)	0.66		
Golimumab	0.05 (0.00–13.94)	0.29		
Secukinumab	0.05 (0.00–282.43)	0.49		
Ustekinumab	0.05 (0.00–657,547)	0.72		

*SpA* Spondyloarthritis; *CI* Confidence interval; *IBD* Inflammatory bowel disease; *DM* Diabetes mellitus; *TB* Tuberculosis; *CKD* Chronic kidney disease; *CLD* Chronic lung disease; *CHD* Chronic heart disease; *CVA* Cerebrovascular accident

### Missing values

Data on smoking and alcohol use were missing in 51 (1.7%) patients, which was not statistically significant.

## Discussion

Patients with SpA had higher crude incidence rates of TB than the general population. Risks factors included age, alcohol use, history of TB, chronic lung disease, duration of glucocorticoid therapy greater than 6 months, and infliximab therapy. The most common site of TB was pulmonary.

Age and sex adjusted crude incidence rates of TB were greater in SpA than in the general population, especially in females. Similarly elevated rates occurred regardless of DMARD therapy, suggesting that drugs were the not sole contributors to increased risk. In contrast, a Swedish study found increased risk of TB in biologics-exposed, compared with biologics-naïve patients with SpA (HR 7.5; 95% CI 1.9–29) [22]. However, prevalence of TB in Asia is much higher than in Europe. The Swedish registry recorded a total of 11 cases of TB in a combined group of 38,702 patients with SpA and 200,417 persons in the general population while this study alone reported 43 cases of TB in 2984 patients with SpA. Data from this study paints a more accurate picture reflecting the endemic burden of TB in Hong Kong [11].

Infliximab was the only DMARD and TNFi found with significantly higher risk of TB in this study. This result is unlikely related to the pre-biologic screening policy for latent TB as most of the cases were recorded after its implementation in 2001 [23]. The highly variable risk profiles of individual DMARDs reflects differences in pharmacodynamic and pharmacokinetic mechanisms. TNFi deactivates T cells and macrophages [24] and induces apoptosis in key immune cells [25]. Infliximab specifically has wider fluctuations in serum levels [26] and higher peak drug concentrations [27] than other TNFi. Changes in levels of TNF-α, associated with maintenance of granuloma integrity, is correlated with disease susceptibility both in experimental models and in humans [28, 29]. It is postulated that differential risks amongst individual TNFi is a result of differences in membrane TNF activation and the resulting effector T cell cascade. Infliximab and adalimumab possess at least 3 to 4 times greater risk of TB than etanercept [30]. A nation-wide South Korean study found the highest risk with infliximab (incidence rate ratio [IRR]: 6.8), followed by adalimumab (IRR: 3.5) and etanercept [6].

Secukinumab or ustekinumab therapy had no association with TB, which is reassuring in this endemic region. Negligible risk in b-DMARDs with the exception of TNFi were cited in many controlled trials [31–33], national registries of biologics [33, 34] and post-marketing surveillance [33]. No cases of TB were found in the Psoriasis Longitudinal Assessment and Registry (PSOLAR) of 3474 patients with psoriasis and PsA given ustekinumab, over a median follow-up of 1.6 years [34], and from pooled safety analysis of 10 studies in psoriasis [33], and one study in AS [35]. Screening for latent TB have led to relatively low rates of reactivation of TB in AS [36]. Mandatory screening for and isoniazid treatment of latent TB prior to starting biologics reduced its occurrence in SpA [37]. Similar guidelines from the Hong Kong Society of Rheumatology have been adopted for many years [15].

Previous history of TB significantly increased the risk of TB reactivation or reinfection, with an HR of 13.88 (95% CI 6.07–31.72) in our study. TB reactivation is a major health concern [38] in patients with HIV or other immunocompromised states in Western countries. However, in highly endemic regions, exposure to TB plays a more important role, while HIV takes a back seat. The prevalence of HIV in Hong Kong is negligible, with 9091 cases in a population of 7.4 million in 2017 [39], as reflected in this study in which no cases of HIV infection were reported.

Other risk factors for TB have been investigated in our cohort. While the risk of TB was no different in psoriasis as in IBD, more TB was observed in younger age groups, which could reflect a migrant population with more diverse social contacts [40]. Unsurprisingly, TB was increased in smokers and patients with chronic lung disease in our study, likely due to ventilatory restriction and impaired lung function [41], and consistent with data in TB endemic areas [42]. Alcohol use was linked to TB in this study, consistent with previous studies which found alcohol misuse contributed to additive risks in current and past smokers [43]. The link between glucocorticoid therapy and TB in our study has also been well established in a number of rheumatologic [44, 45] and non-rheumatologic conditions [46]. As long-term glucocorticoid therapy is not recommended in international consensus statements [47, 48], rheumatologists should be prudent in considering options for drug treatments.

Sites of TB infection may reflect the magnitude of immunosuppression, either from the disease process itself or immunosuppressive drugs. Extrapulmonary TB represents reactivation rather than nascent infection as mycobacteria from the encapsulated granuloma in the lung spread to other sites via the blood or lymphatic system [49]. In our cohort, extrapulmonary TB occurred in 4 (9.3%) out of 43 patients with SpA, less than in RA [50], likely reflecting reduced immunosuppression from lower cumulative exposure to DMARDs. Screening and chemoprophylaxis may also have decreased TB reactivation.

### Limitations and future direction

Small size of the group with TB contributed to potential bias. Limited data from subgroups of newer drugs like secukinumab and usterkinumab should be interpreted with caution. Disease activity and chronicity, which may affect the risk of TB, were not included in this study. Future studies should include prospective multinational registries to strengthen surveillance of TB [51].

## Conclusion

The crude incidence rate of TB was increased in SpA when compared to the general population. Independent risk factors for TB were alcohol use, previous TB, chronic lung disease, history of ischemic stroke, glucocorticoid therapy and infliximab therapy. Biologics with the exception of infliximab should be considered in SpA patients at risk for TB.

## Data Availability

Data is available from Dr. Ho Yin Chung upon reasonable request.

## Abbreviations

TB: Tuberculosis
SpA: Spondyloarthritis
NSAID: Non-steroidal anti-inflammatory drug
bDMARD: Biological disease modifying anti-rheumatic drug
cDMARD: Conventional disease modifying anti-rheumatic drug
AS: Ankylosing spondylitis
PsA: Psoriatic arthritis
IBD: Inflammatory bowel disease
uSpA: Undifferentiated spondyloarthritis
HLA: Human leukocyte antigen
TNFi: Tumor necrosis factor inhibitor
CMS: Clinical management system
DM: Diabetes mellitus
CKD: Chronic kidney disease
CLD: Chronic lung disease
CHD: Chronic heart disease
CVA: Celebrovascular disease
CI: Confidence interval
HR: Hazard ratio
HIV: Human immunodeficiency virus
IRR: Incidence rate ratio
PSOLAR: Psoriasis longitudinal assessment and registry
